# Evaluating Pretrained Protein Language Model Embeddings as Proxies for Functional Similarity

**DOI:** 10.1007/s00239-025-10282-4

**Published:** 2025-11-22

**Authors:** Robert Shaw, Samuel D. Love, Claire D. McWhite

**Affiliations:** Department of Molecular and Cellular Biology, The University of Arizona, Tucson, AZ USA

**Keywords:** Protein language models, Functional similarity

## Abstract

Protein Language Models (PLMs) have emerged as powerful tools for representing protein sequences. We explore how embeddings (numeric vector representations) from pretrained PLMs can serve as direct numeric proxies for protein structure and function without requiring additional training or fine-tuning. In a proof-of-concept study of 22 cross-species complementation triplets—a gold standard for functional similarity where genes from one species are tested for their ability to rescue gene deletions in another species—we find that ESM-C 600 M embeddings summarized into pooled sliced-Wasserstein embeddings achieved high discrimination of subtle functional differences. This pooling method captures distributional properties of amino acid embeddings by comparing them against reference points using optimal transport theory. While our limited sample size precludes definitive conclusions about whether PLM embeddings systematically outperform sequence-based methods in detecting protein functional similarity, our preliminary results demonstrate the potential of using protein embeddings for functional analysis. Our exploratory analysis of orthology relationships suggests that embedding similarity may correlate with functional conservation, with the least diverged ortholog showing higher embedding similarity in approximately two-thirds of cases. Analyzing the Ortholog Conjecture—that orthologs maintain greater functional similarity than paralogs at equivalent sequence divergence—we do not observe clear differences between one-to-one orthologs and inparalog embedding similarities. Finally, we propose integrating PLMs with phylogenetic methods in a hybrid approach that leverages their complementary strengths: PLM-derived numeric embeddings for rapid homology detection and phylogenetics for evolutionary precision. We introduce embedding-tree versus gene-tree discordance as a potential metric to detect functional divergence between closely related proteins. Integrating protein embeddings with sequence analysis may enable a more nuanced understanding of protein function and evolutionary dynamics.

**Supplementary Information:**

The online version contains supplementary material available at 10.1007/s00239-025-10282-4.

## Introduction

The relationship between protein sequence and function is a fundamental challenge in biology (Redfern et al. 2008; Whisstock and Lesk 2003; Park et al. 2024). Protein language models (PLMs) have recently emerged as powerful tools for analyzing this sequence-function relationship (Bepler and Berger 2021; Rives et al. 2021; Lin et al. 2023). Trained on vast databases of protein sequences, these models have demonstrated remarkable success in predicting protein structure and function. This ability to convert protein sequences into highly meaningful numerical representations using language models has revolutionized computational biology, enabling advances in protein structure prediction (Lin et al. 2023; Wu et al. 2022), designing novel proteins (Madani et al. 2023; Sgarbossa et al. 2023), predicting drug-target interactions (Ünlü et al. 2025), engineering antibodies (Hie et al. 2024), and many other applications.

**Fig. 1:**
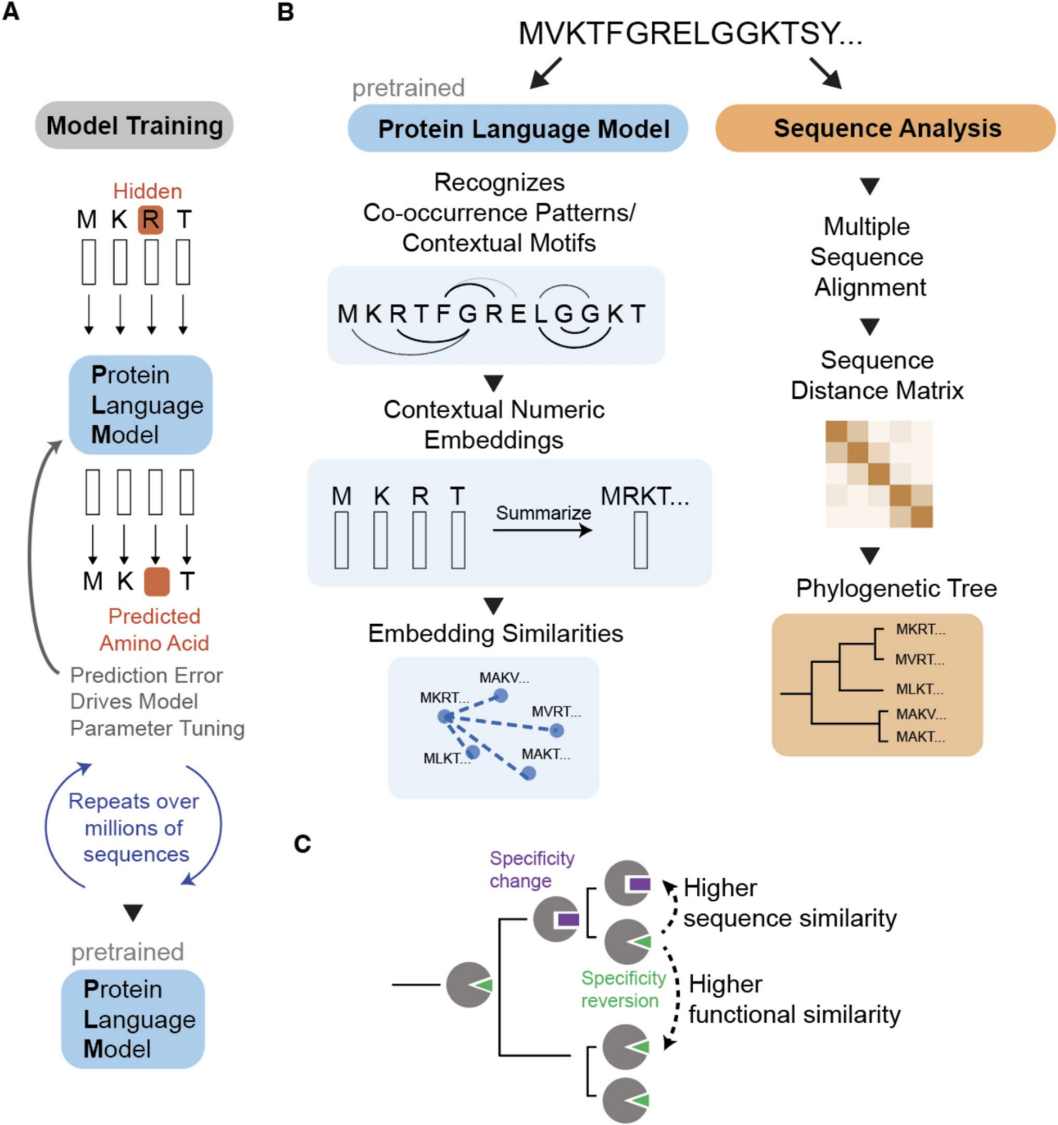
A comparison of how protein language models and phylogenetic methods process protein sequences differently. **A** In one training method, PLMs are trained to predict masked or hidden amino acids in protein sequences, which tunes model weights to recognize sequence patterns. **B** The left panel shows how PLMs convert protein sequences into embeddings that capture functional relationships, clustering proteins based on structural and functional similarities in high-dimensional space. The right panel illustrates how phylogenetic methods align sequences to reconstruct evolutionary history, organizing proteins in trees based on shared ancestry and divergence. **C** Sequence similarity indicates evolutionary relatedness, while functional similarity captures biochemical properties. For example, two enzymes may have similar catalytic specificities despite different evolutionary origins

Protein language models are typically transformer-based neural networks that learn to predict hidden amino acids in a sequence based on their context (Fig. 1A). This self-supervised training objective enables the model to learn meaningful representations of sequences without requiring labeled data (Vaswani et al. 2017; Elnaggar et al. 2022). A model that has undergone this initial training process is called a “pretrained” model.

During pretraining, the model processes millions of protein sequences, learning which amino acid patterns commonly co-occur. The key insight is that these models learn the “grammar” of protein sequences—the rules that govern which amino acid combinations can create biologically realistic proteins. This is analogous to how other language models learn that certain word combinations make semantic sense while others do not. Just as a standard language model might learn that "the cat meows" is more likely than "the cat photosynthesizes," a protein language model learns that certain amino acid combinations and arrangements are more likely to exist than others. Consider a zinc finger CCHH motif: when given the sequence containing the motif CC[hidden]H, a language model can learn that this pattern highly predicts that the hidden token is H. An attention head responsive to this particular motif was identified in Vig et al. (2020). Beyond linear/local motifs, Zhang et al. (2024) demonstrated that LLMs additionally capture long-range motifs between pairwise contacts, which is a data structure that underlies their utility for protein folding tasks.

Protein language models process protein sequences by converting them into embeddings (vectors of numbers) that capture the essential properties of the input sequence, representing complex patterns in a high-dimensional space. For example, when a sequence like “MCKTFGRELGGKTSY” passes through a PLM (Fig. 1B, left panel), the model generates a vector of numbers for each amino acid based on identified sequence patterns. These embeddings have been shown to reflect biological properties such as structural tendencies, functional domains, and biochemical characteristics (Vig et al. 2020). The amino-acid level embedding vectors can then be summarized to produce a single embedding for a protein, and then used to measure similarity between proteins or serve as inputs for classical machine learning algorithms. The result is that functionally related proteins cluster together in the high-dimensional embedding space based on their shared structural and functional motifs. However, PLMs will inevitably also learn sequence patterns that reflect the underlying phylogenetic imbalances and sampling biases in the sequence databases they are originally trained on (Weinstein et al. 2022).

While PLMs effectively capture patterns in sequence data, they do not explicitly model the historical processes that produced those sequences, such as mutations, selection, and speciation events (Elnaggar et al. 2022). This contrasts with phylogenetic methods, which are specifically designed to infer evolutionary relationships and reconstruct the historical processes that gave rise to observed sequence data (Fig. 1B, right panel).

The relationship between functional similarity and evolutionary history is complex (Fig. 1C). Proteins with similar structures and functions are often evolutionarily related, but not always. Convergent evolution can lead to similar structures/functions in relatively distantly related proteins, while gene duplication followed by divergence can lead to different functions in closely related proteins. This complexity creates an interesting intersection between what PLMs capture (primarily functional/structural patterns) and what phylogenetic methods capture (primarily evolutionary relationships). This fundamental difference leads to a key insight: sequence similarity informs on evolutionary relatedness and only indirectly reflects functional similarity, while embedding similarity captures shared patterns within sequences and only indirectly correlates with evolutionary relationships.

Currently, pretrained PLMs have significant advantages over sequence-based methods at remote distances, beyond the “twilight zone” of sequence similarity. Our findings align with broader patterns observed in PLM performance across different evolutionary distances. Multiple studies have demonstrated that PLM embeddings excel at capturing broad protein classes and remote homology (Tule et al. 2025; Flamholz et al. 2024; Liu et al. 2024). For highly diverged protein families, we have demonstrated that PLMs enable more accurate sequence alignments than traditional approaches by capturing positional and contextual information that allows better position matching even when amino acids are poorly conserved (McWhite et al. 2023). Tule et al. (2025) found that pretrained PLM embeddings accurately match phylogenetic distances for broad evolutionary relationships within PFAM families but are less accurate for resolving relationships between closely related proteins. This pattern reflects the underlying biology of protein evolution and PLM training. At broader evolutionary distances, functional constraints dominate sequence signals—only functionally essential features are conserved across large evolutionary spans. These are precisely the patterns that PLMs learn effectively from their training data. At shorter evolutionary distances, neutral or nearly neutral mutations that don’t substantially affect function can accumulate, creating evolutionary signals that PLMs may not capture since they do not explicitly model evolutionary processes.

Protein embeddings from protein language models have the potential to enable direct study of functional similarity from sequences, even without additional training. Since many biological problems lack sufficient data for fine-tuning, pretrained embeddings could potentially serve as numeric proxies for proteins, as they capture patterns within protein sequences. However, the suitability of embeddings from pretrained PLMs for distinguishing subtle functional differences between proteins has not been directly assessed.

Ideally, an embedding from a pretrained PLM could serve as a numeric proxy for a protein’s structure and function. For the purposes of this paper, we define function as the biochemical activity of a protein, which is determined by its three-dimensional structure and the physical properties arising from this structure. This includes the protein’s ability to interact with other molecules, its catalytic activity, and its contribution to cellular processes, all of which are guided by the specific sequence and conformation of the proteins (Sadowski and Jones 2009). We consider two proteins to be functionally near equivalent (Koonin 2005) if swapping the two proteins between their species-of-origin would cause no biochemical differences or other effects.

PLM embeddings are typically used in three primary ways: as direct numeric proxies without additional training (one-shot), as input features for supervised learning, or through fine-tuning for specific tasks (Rao et al. 2019; Heinzinger et al. 2022; Elnaggar et al. 2022; Lin et al. 2023). While many of the most powerful demonstrations of PLMs involve further training or fine-tuning, a clear demonstration that pretrained embeddings alone can capture subtle functional differences between proteins is lacking in the literature. This is a critical gap, as biological research typically involves small-scale studies of specific protein families rather than the large labeled datasets that supervised learning requires, making pretrained embeddings that intrinsically capture functional properties especially valuable.

**Fig. 2:**
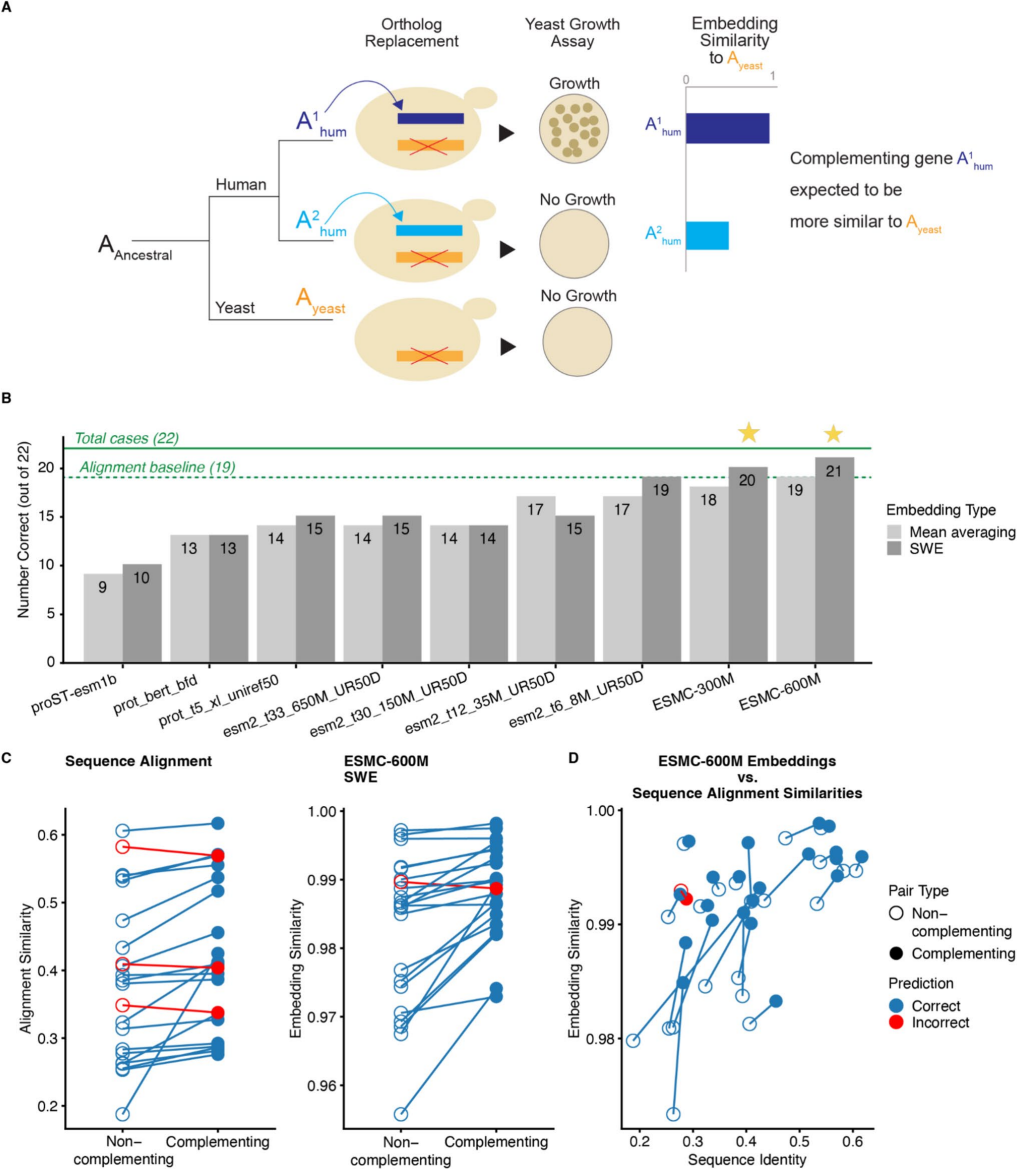
Comparison of different protein language models for predicting cross-species complementation between proteins. **A** In cross-species complementation experiments, the ability of orthologous human genes to complement deleted yeast essential genes is tested. We expect the complementing human gene to have higher embedding similarity to the yeast gene than the non-complementing human gene. **B** Bar chart showing the predictive accuracy of various protein language models in identifying which human or Arabidopsis paralog can functionally complement a yeast ortholog. The horizontal line indicates baseline accuracy using sequence identity (19/22 correct). Smaller models (e.g., ESM2 8 M) outperform larger models (e.g., ESM2 150 M and 650 M), and pooled sliced-Wasserstein embeddings (SWE) improve performance across models, with ESM-C models showing the highest accuracy. **C** Scatter plot of sequence identity vs. ESM-C 600 M embedding similarities, with complementing pairs (filled circles) and non-complementing pairs (open circles) showing how embeddings can potentially distinguish functional relationships even when sequence similarities are ambiguous

The difficulty in demonstrating this capability stems from the lack of large-scale datasets of closely related protein sequences with known functional differences. While a large number of functional annotations exist for proteins, differences in annotation between homologous proteins does not necessarily mean differences in function and may instead reflect annotation bias (Thomas et al. 2012). We thus focus on cross-species complementation as a gold-standard source of true functional differences between proteins. In these experiments, a gene from one species is used to complement the deletion of a gene from another species, providing a natural validation of functional similarity (Fig. 2A). Failure of a protein to complement its ortholog in another species indicates that some level of functional divergence has occurred between the two proteins. We use cross-species complementation as a strict evaluation benchmark rather than for training, as examples are limited.

In this article, we first demonstrate through cross-species complementation experiments that pretrained**/**”naive” embeddings may be able to capture subtle functional differences between proteins better than sequence similarity. While these cross-species complementation examples are limited in number, they represent a gold standard assessment of protein functional similarity and dissimilarity. We additionally evaluate diverse models and embedding strategies to better capture functional differences, analyze orthology relationships to understand when embedding and sequence similarity align or diverge, and suggest integrating PLMs with phylogenetic methods in a hybrid approach that leverages their complementary strengths.

## Results

### Evaluating PLMs Potential Ability to Detect Subtle Functional Differences Between Proteins Using Cross-species Complementation Assays

Laurent et al. (2020) performed cross-species complementation yeast humanization experiments (Laurent et al. 2015; Kachroo et al. 2015), where *Saccharomyces cerevisiae* genes were deleted, and the ability of corresponding human proteins to complement the deletion was tested. In one experiment, yeast genes with more than one ortholog in humans were replaced with one of these human orthologs at a time. In some cases, only one of the two human orthologs was able to complement the deletion, indicating that one human ortholog is more diverged in function from the yeast protein than the other (Fig. 2A). Garge et al. (2020) continued this effort by testing complementation of yeast genes with multiple human orthologs. Kachroo et al. (2017) additionally test the ability of several genes from *Arabidopsis thaliana* to complement steps in heme biosynthesis.

We use these non-complementing/complementing pairs to explore the ability of different pretrained embeddings to reflect subtle functional differences between paralogs. Critically, the complementing human protein should be functionally more similar to the yeast protein than the non-complementing human ortholog, as it can successfully perform the biological role of the corresponding yeast protein. If PLM embeddings truly capture functional relationships, then the complementing human protein should have higher embedding similarity to the yeast protein than the non-complementing human ortholog (Fig. 2A). These experiments provide a direct test of whether pretrained PLM embeddings truly capture functional similarity between proteins.

Sequence similarity is already quite good at this task. In cases where one species has a single gene copy while another species has multiple copies of that gene, the Least Diverged Ortholog (LDO) refers to the copy showing the greatest sequence similarity to the single-copy gene (Mi et al. 2010). The LDO (based on sequence identity) is the complementing paralog in 19 out of 22 cases. However, sequence identity gives us a baseline to test whether PLM embeddings can do even better at capturing the functional relationships that determine complementation success. For the following analyses, we calculated sequence identity as the fraction of identical amino acid positions in the aligned sequences.

To evaluate the broader landscape of PLM performance, we tested a range of models specifically designed for representation learning including different sizes of the ESM2 series (8 M, 35 M, 150 M, 350 M, 650 M parameters) (Lin et al. 2023), prot_bert_bfd (Elnaggar et al. 2022), prot_t5_xxl_uniref50 (Elnaggar et al. 2022), the ESM-C 300 M and 600 M parameter models (ESM Team 2024), and the multimodal model ProtST (Xu et al. 2023). For ESM-C, we specifically used the ESMplusplus-small (300 M) and ESMplusplus-large (600 M) implementations (Hallee et al. 2024), which are compatible with the Huggingface (Wolf et al. 2020) infrastructure and do not substantially differ from the original ESM-C models. All models were accessed through the Huggingface framework (Wolf et al. 2020).

To obtain a single embedding for a protein sequence, the amino acid-level embeddings produced by PLMs must first be summarized into a single sequence-level embedding. The standard practice is to average amino acid-level embeddings from the final layer (Vieira et al. 2025), but this approach can be affected by protein length differences. We instead used sliced-Wasserstein embeddings (SWE) (NaderiAlizadeh and Singh 2025), which takes a different approach based on optimal transport theory. SWE treats the amino acid-level embeddings from each protein as an empirical probability distribution, and compares each protein’s distribution against a set of fixed reference points (landmark positions in embedding space). SWE projects both the protein embeddings and reference points onto multiple one-dimensional slices, where optimal transport distances can be calculated efficiently. The resulting pattern of distances to the reference points becomes a fixed-length representation of the protein, capturing more information about the embedding distribution than simple averaging. We used SWE with randomly initialized and frozen parameters (both projection directions and reference points), and instead of learning combination weights as in the original framework, we used simple mean averaging to pool fixed-length SWE representations into a single sequence embedding.

For each model, we generated embeddings of the yeast proteins and their human orthologs using both standard mean averaging and pooled SWE, then used cosine similarity to predict which human ortholog would functionally complement the yeast protein (Supplemental File1, Fig. 2B).

We tested using embeddings from the final layer, the last 4 layers, and the last 8 layers of all models (Supplemental Figure 2). The results for the final 8 layers are displayed in Fig. 2B.

The results across models revealed several surprising patterns. Interestingly, the mistakes made by language models and sequence similarity do not overlap. For example, all PLMs and embedding approaches tested are able to predict that CDS2_HUMAN will complement CDS1_YEAST, even though CDS1_HUMAN is the Least Diverged Ortholog (at 37.85% identity vs. 35.14% for CDS2_HUMAN).

Across models, pooled SWE representations were generally better at distinguishing complementing from non-complementing paralogs than standard mean pooled embeddings (*p* = 0.0049, Wilcoxon signed-rank test). Interestingly, smaller ESM2 models were better able to distinguish complementing from non-complementing proteins than larger ones. Pretrained pooled SWE representations from the 350 M parameter ESM2 model only predicted 15 out of 22 complementing/non-complementing protein pairs correctly, while pooled SWE representations from the substantially smaller 8 M parameter model predicted 19 out of 22 correctly (Fig. 2B). This pattern is not unique to our complementation task. Similar observations where smaller models outperform larger models have been made for several other applications directly using pretrained outputs and embeddings from PLMs in the context of zero-shot variant effect prediction (Cheng et al. 2024; Hou et al. 2025; Nijkamp et al. 2023).

Overall, pooled SWE representations from the ESM-C 600 M parameter model showed the best ability to distinguish subtle functional differences between closely related proteins in our small dataset. This model and embedding approach achieved 21 out of 22 correct classifications of complementing vs. non-complementing paralog pairs (Fig. 2B). Pooled SWE representations of ESM-C 300 M were the second most accurate, with 20/22 correct classifications. However, the highest accuracy of ESM-C 600 M (21/22) compared to the alignment baseline (19/22) is not statistically significant due to the small sample size. Figure 2C plots the sequence identities and ESM-C 600 M embedding similarities for all 22 non-complementing/complementing pairs. There is a clear trend of increasing accuracy with more recently released models, suggesting ongoing improvements in embedding quality.

While the embeddings showed better classification than sequence similarity alone (which correctly predicted 19 out of 22 cases), the small sample size limits our ability to draw definitive conclusions about the correspondence between embedding similarity and functional similarity, and will require future validation with larger datasets.

Notably, the single case where ESM-C 600 M made an incorrect prediction was marginal, where the embedding similarities between the complementing and non-complementing proteins differed by only slightly, as shown in Fig. 2D. This near-tie suggests that as PLMs continue to improve, even these borderline functional differences may become reliably detectable. As results for the different layer choices do not appear to substantially vary for ESM-C 600 M, subsequent analyses using this model use only the final layer.

Additionally, the potential accuracy of PLMs embeddings in prioritizing complementing proteins appears to be currently limited to pairs of paralogs. For one-to-many orthology relationships tested in Laurent et al. (2020) and Garge et al. (2020), where yeast has one gene and humans have many inparalogs (specifically β-tubulin, Actin, Myosin, and Uroporphyrinogen decarboxylase), both sequence identity and ESM-C embeddings are poor predictors of which of the human paralogs will complement the yeast deletion (Supplemental Figure 1). We speculate that in expanded orthogroup families, the more complex patterns of sub- and neofunctionalization found in multiparalog expansions (Voordeckers et al. 2012) may obscure which human paralog is able successfully complement the yeast function.

### Sequence Identity and Embedding Similarity Concordance

We next examined how embedding similarities relate to evolutionary patterns across larger datasets. While the limited (n = 22**)** cross-species complementation experiments provide initial support for the functional relevance of these embeddings, the broader patterns we observe should be interpreted as preliminary findings that may evolve as PLM architectures and pretraining methods continue to advance. Additionally, the limited statistical power of our complementation analysis (n = 22) warrants caution in extrapolating these findings to broader patterns of protein functional relationships.

**Fig. 3:**
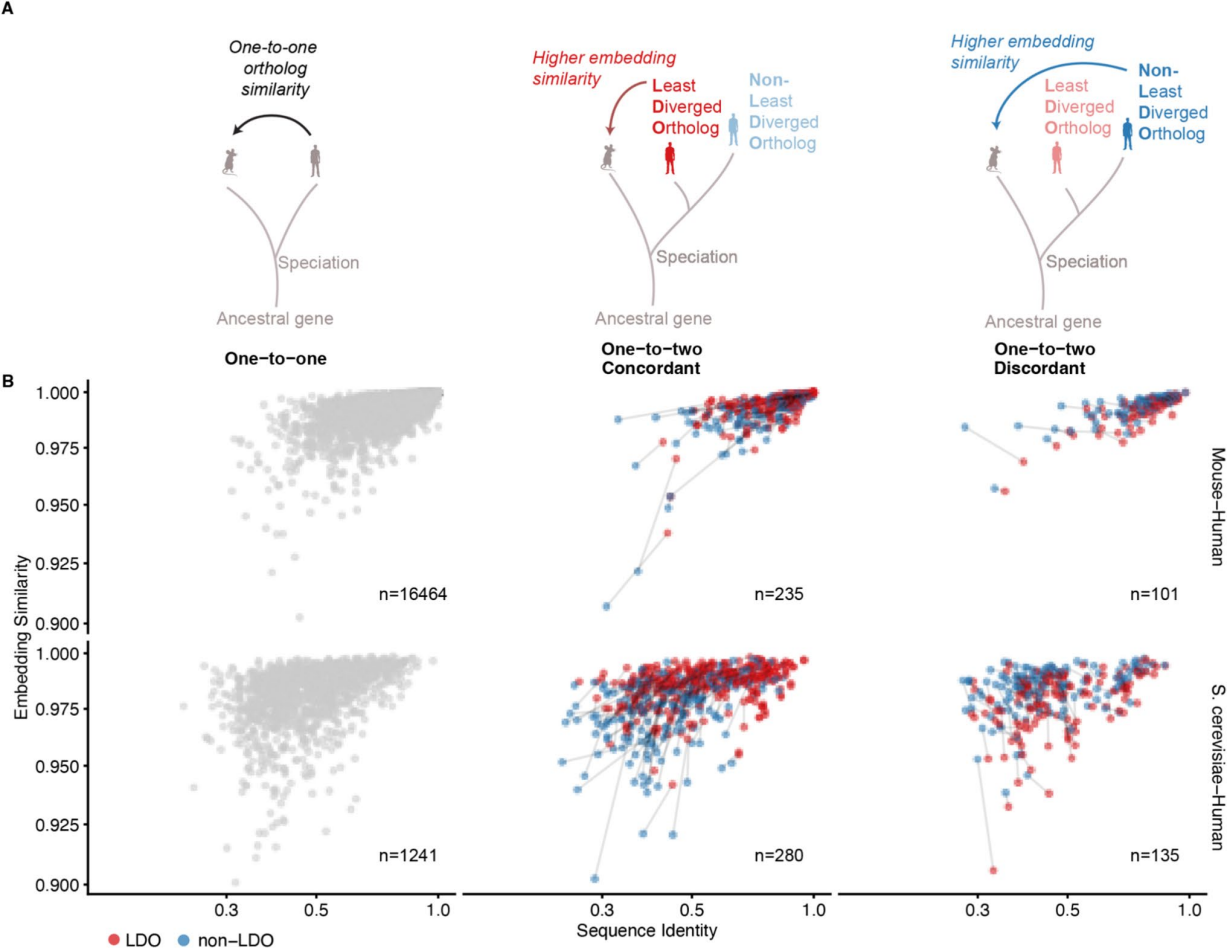
Sequence identity versus embedding similarity for LDO and non-LDO proteins. **A** Schematic illustration of the Least Diverged Ortholog (LDO) concept in one-to-two orthology relationships, where the ortholog with highest sequence similarity to the single-copy protein is defined as the LDO. Both concordant and discordant cases are illustrated. **B** Scatter plots comparing embedding similarity versus sequence identity for ortholog relationships between human-mouse and human-S. cerevisiae. The plots are divided into three categories: one-to-one relationships (left column), one-to-two relationships with expected concordance between sequence and embedding similarity (middle column), and one-to-two relationships with unexpected discordance between sequence and embedding metrics (right column). Points connected by lines represent inparalog pairs, with approximately two-thirds showing concordance between sequence identity and embedding similarity

Given that ESM-C 600 M demonstrated superior performance in our complementation analysis, we used its embeddings to explore orthology relationships between human and other species. For one-to-many ortholog relationships, where species A has a single copy of a gene and species B has more than one copy, the Least Diverged Ortholog (LDO) is defined as the ortholog with highest sequence similarity to the single-copy protein (Mi et al. 2010) (Fig. 3A). We surveyed all one-to-two orthology relationships between human and *S. cerevisiae*, and between human and mouse, to determine how often the LDO (based on sequence identity) also shows the highest embedding similarity. Ortholog and paralog relationships between three species: Homo sapiens (9606), Mus musculus (10,090), and Saccharomyces cerevisiae (559,292) were downloaded from InParanoiDB 9 (Persson and Sonnhammer 2023).

We divided inparalog pairs into “concordant” cases where the protein with the highest sequence identity also has the highest embedding similarity, and “discordant” cases where embedding similarity and sequence identity disagree (Fig. 3A). In both *S. cerevisiae*-human and mouse-human comparisons, approximately two-thirds of inparalog pairs showed concordance between embedding similarity and sequence identity (Fig. 3B).

With the caveat that more confidence must first be gained in the correspondence between embedding similarity and functional similarity, the one-third of cases showing discordance could represent particularly interesting examples where functional constraints have evolved differently than overall sequence similarity would predict. These discordant cases highlight the potential value of using both evolutionary and functional approaches to understand protein relationships.

### Testing the Ortholog Conjecture

The ortholog conjecture states that orthologs tend to be more functionally similar than paralogs at the same level of sequence divergence (Koonin 2005). This hypothesis has been difficult to test comprehensively due to the challenge of quantifying functional similarity across many proteins. Functional annotations are often biased toward highly studied genes and reflect species-specific experimental focus rather than actual functional differences. For example, Gene Ontology (GO) annotations for orthologous genes often reflect differences in experimental priorities between species rather than true functional divergence. (Thomas et al. 2012) As a result, studies have turned to alternative proxies such as RNA expression data (Rogozin et al. 2014; Chen and Zhang 2012) and careful examination of experimentally-confirmed functional differences (Altenhoff et al. 2012) to test the ortholog conjecture. With function-aware embeddings that reliably reflect functional properties, there would be an opportunity to test this conjecture at scale using a different approach that may avoid some of these traditional limitations.

**Fig. 4:**
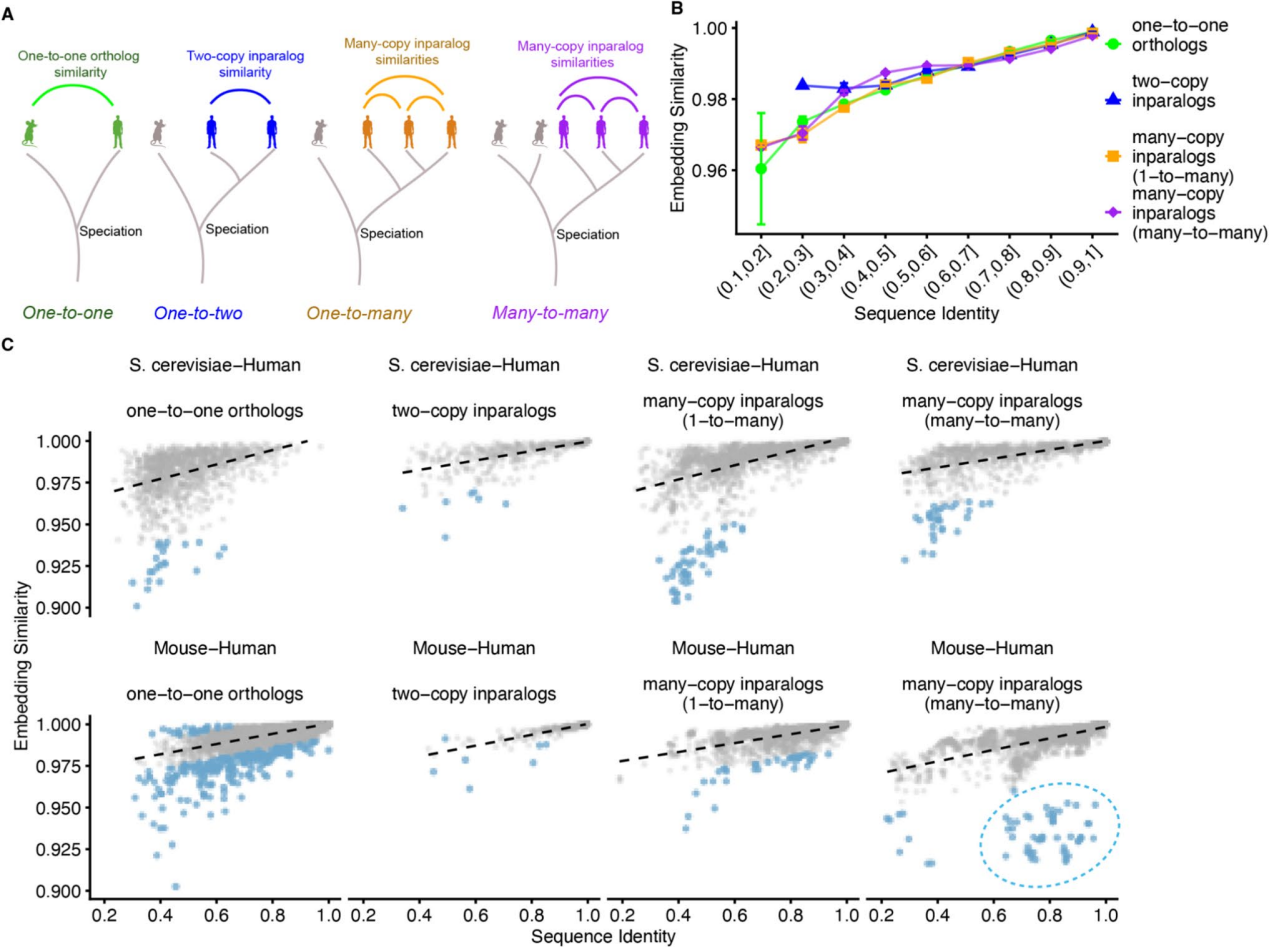
Testing the ortholog conjecture using protein embeddings. **A** Schematic of orthology and inparalogy relationships. **B** Analysis of embedding similarity across different orthology relationship categories, with one-to-one orthologs, two-copy inparalogs, and multi-copy inparalogs from one-to-many and many-to-many relationships plotted against sequence identity bins. **C** Detailed scatter plots comparing embedding similarity versus sequence identity across different orthology categories for S. cerevisiae-human (top row) and mouse-human (bottom row) protein relationships. Points further than 3 standard deviations from the linear fit are colored in light blue. Notable outliers are circled in the mouse-human many-to-many category include immunoglobulin variable domains, which show lower embedding similarity than expected from their sequence identity

It is important to note that when multiple human paralogs exist for a single yeast gene (one-to-many relationships), PLM embeddings showed much weaker performance at identifying the complementing protein in cross-species complementation experiments (Supplemental Figure 1). Embedding similarity alone may not be the correct metric to capture the functional partitioning that arises from subfunctionalization following multiple gene duplications. We thus compared embedding similarity of one-to-one orthologs with two-copy inparalogs, multi-copy inparalogs deriving from one-to-many relationships, and multi-copy inparalogs deriving from many-to-many relationships (Fig. 4A) using ESM-C 600 M embeddings. In bulk, there was no clear difference in embedding similarity across these groups (Fig. 4B). We next plotted sequence identity versus embedding similarity for these categories for *S. cerevisiae*-human and mouse-human comparisons (Fig. 4C). Ortholog and inparalog pairs falling more than 3 standard deviations away from the line-of-best-fit for each category are highlighted in light blue. Across all ortholog and inparalog relationships, we observe a region of these outliers where embedding similarity is lower than would be expected by sequence similarity (Fig. 4C). For example, among the many-copy paralogs between human and mouse the variable domains of immunoglobulin light chains (Ig kappa chain V-III) (Fig. 4C, blue dashed circle) showed much lower embedding similarity than predicted by sequence identity, capturing the fact that these proteins are experiencing functional change faster than their sequence identities would predict.

This example illustrates a key strength of PLM embeddings: they can identify cases where functional constraints are evolving rapidly despite relatively conserved sequences. The immunoglobulin variable domains are under strong diversifying selection for antigen recognition, leading to functional divergence that exceeds what sequence similarity alone would suggest. As embeddings become more representative of protein function, they may prove increasingly useful for detecting such cases where evolutionary and functional signals diverge.

## Discussion

The true utility of PLM-derived embeddings for studying and annotating protein function at scale will be unlocked when we can gain confidence that an embedding is a faithful reflection of true functional and structural properties of proteins. Such representations would enable meaningful analyses of protein sequences without requiring specialized training datasets and fine-tuning for each new research question.

A function-aware embedding should be responsive to mutations in such a way that mutations perturb the embedding in ways that reflect actual functional changes in the protein. Proteins with more similar physical properties should have more similar embeddings. Our analysis of a limited set of 22 cross-species complementation experiments provides early evidence that current embeddings from pretrained models, particularly from ESM-C, are moving in this direction. Better pooling of amino acid embeddings into representative sequence embeddings, for example through using averaged sliced-Wasserstein embeddings (NaderiAlizadeh and Singh 2025) broadly improves the ability to distinguish complementing and non-complementing paralogs across multiple protein language models.

However, important limitations remain, particularly in predicting complementation for highly expanded protein families. While our functional exploration is based on 22 complementation experiments, this reflects the scarcity of such high-quality functional datasets. Cross-species complementation experiments with differential replaceability (where some orthologs complement while others do not) represent one of the most stringent tests of protein functional similarity available, making datasets like those from Laurent et al. (2020) and Garge et al. (2020) exceptionally valuable. Larger-scale functional datasets of this caliber will enable even more comprehensive validation of computational approaches to identify subtle functional differences.

There is a fundamental difference between sequence similarity and embedding similarity: sequence similarity informs on evolutionary relatedness and only indirectly reflects functional similarity, while embedding similarity captures shared patterns within sequences and only indirectly correlates with evolutionary relationships. The language model, focused on learning amino acid patterns and predicting hidden amino acids based on context (Elnaggar et al. 2022), has no mechanism for tracking the historical sequence of mutations that led to current protein sequences. The sequence patterns that PLMs learn are predominantly structural, including conservation (Brixi et al. 2025; Yeung et al. 2023; Marquet et al. 2022), co-evolution (Zhang et al. 2024), and functional motifs (Vig et al. 2020). This creates substantial informational overlap between PLM embeddings and protein structures, underlying the extraordinary success of PLMs in predicting protein structure (Zhang et al. 2024). The fact that structural patterns are more conserved than sequence (Chothia and Lesk 1986) additionally underlies the superior capabilities of both PLM embeddings and structure to build phylogenies of sequence diverged proteins (Tule et al. 2025; Moi et al. 2025) and detect homologs (van Kempen et al. 2024; Jumper et al. 2021) at evolutionary distances where sequence-based methods fail (Rost 1999). However, both PLM embeddings and structure are less effective at resolving fine-scale evolutionary relationships (Tule et al. 2025; Moi et al. 2025), as even single amino acid changes can substantially alter both representations, even at high levels of sequence similarity. This all contrasts with phylogenetic methods, which use explicit models of sequence evolution with sophisticated statistical approaches to account for multiple substitutions, rate heterogeneity across sites, and varying selection pressures to reconstruct historical relationships (Yang and Rannala 2012; Whelan et al. 2001).

There is great potential to integrate PLMs, structure, and sequence for orthology that leverages the distinct strengths of each approach. One framework for integration would involve using PLM embeddings for the fast initial grouping of potentially related sequences, followed by traditional phylogenetic methods for fine evolutionary analysis within these groups. This could significantly accelerate orthology detection pipelines while maintaining phylogenetic accuracy. This approach is already being implemented by orthology detection tools like SonicParanoid2 (Cosentino et al. 2024), which incorporates machine learning and language models to accelerate orthology inference.

Following phylogenetic analysis, PLMs could then come back into the picture to aid functional annotation transfer. The vast majority of proteins will never be experimentally characterized, making the propagation of annotations from the few characterized proteins to uncharacterized ones a grand challenge of biocuration (de Crécy-Lagard et al. 2022; Gaudet et al. 2011). PLMs’ ability to capture functional similarity could help determine which annotations are most likely to transfer reliably between orthologs.

There is substantial promise in using phylogenetic information to refine PLM outputs or incorporate phylogenetic sampling during pretraining. Despite efforts to create representative training sets (Suzek et al. 2015), current PLMs inevitably learn from sequence patterns that reflect phylogenetic imbalances and sampling biases in sequence databases. PLM-derived fitness estimates are biased by sequence and species overrepresentation during pretraining (Weinstein et al. 2022), but accounting for phylogenetic structure broadly improves variant effect prediction accuracy (Pugh et al. 2025). This demonstrates both the reality of phylogenetic bias in PLM representations and the value of phylogenetic methods in correcting it. Smaller models may paradoxically avoid some of these biases due to capacity constraints, forcing them to learn only the most generalizable patterns rather than memorizing dataset-specific details including overrepresentation of certain sequence families.

Building on methods established in evolutionary biology for detecting discordance between different phylogenetic signals (Maddison 1997; Stolzer et al. 2012), we propose embedding-tree versus gene-tree discordance as a metric to detect unexpected functional divergence in protein families. In this approach, a gene tree constructed from sequence similarity represents evolutionary relationships, while a second tree derived from PLM embedding similarities captures functional relationships. By quantifying topological differences between these trees (Briand et al. 2020), it may be possible to identify specific proteins where evolutionary history and functional profile significantly diverge beyond expectation. High discordance may indicate cases of functional specialization, convergent evolution, neo-functionalization, or cases where function is not tightly linked to sequence that would benefit from targeted experimental validation.

Continued improvements in PLM architectures and training methods will likely enhance the fidelity of embeddings as functional representations. If embeddings can reliably capture functional properties without requiring task-specific training, they would address a persistent challenge in computational biology: the mismatch between typical biological research questions and the large labeled datasets required for supervised learning approaches. Such developments would make protein analysis more accessible to researchers working on specific protein families or mechanistic questions, where generating extensive training data is often impractical. This integration of protein language models and evolutionary analyses will represent a fundamental shift in the analysis of protein structure, function, and evolution.

## Supplementary Information

Below is the link to the electronic supplementary material.

Supplementary file1 (DOCX 424 kb)

Supplementary file2 (CSV 1087 kb)

## Data Availability

All code used for embedding generation, similarity calculations, data analysis, and figure creation is available at https://github.com/mcwhitelab/plm-complementation.
