# Supplementary material for: Evaluating Pretrained Protein Language Model Embeddings as Proxies for Functional Similarity
Source: J Mol Evol. 2025 Nov 22;93(6):765–76. doi: 10.1007/s00239-025-10282-4 (PMC12756192; doi:10.1007/s00239-025-10282-4)
Supplement: Supplementary file 1 — Supplementary file1 (DOCX 424 kb) [file 239_2025_10282_MOESM1_ESM.docx]

**SUPPLEMENTAL INFORMATION**

Supplemental File 1: Embedding similarities (with and without SWE) and sequence identities for cross-species complementation experiments for all models and layer choices

Supplemental Figures


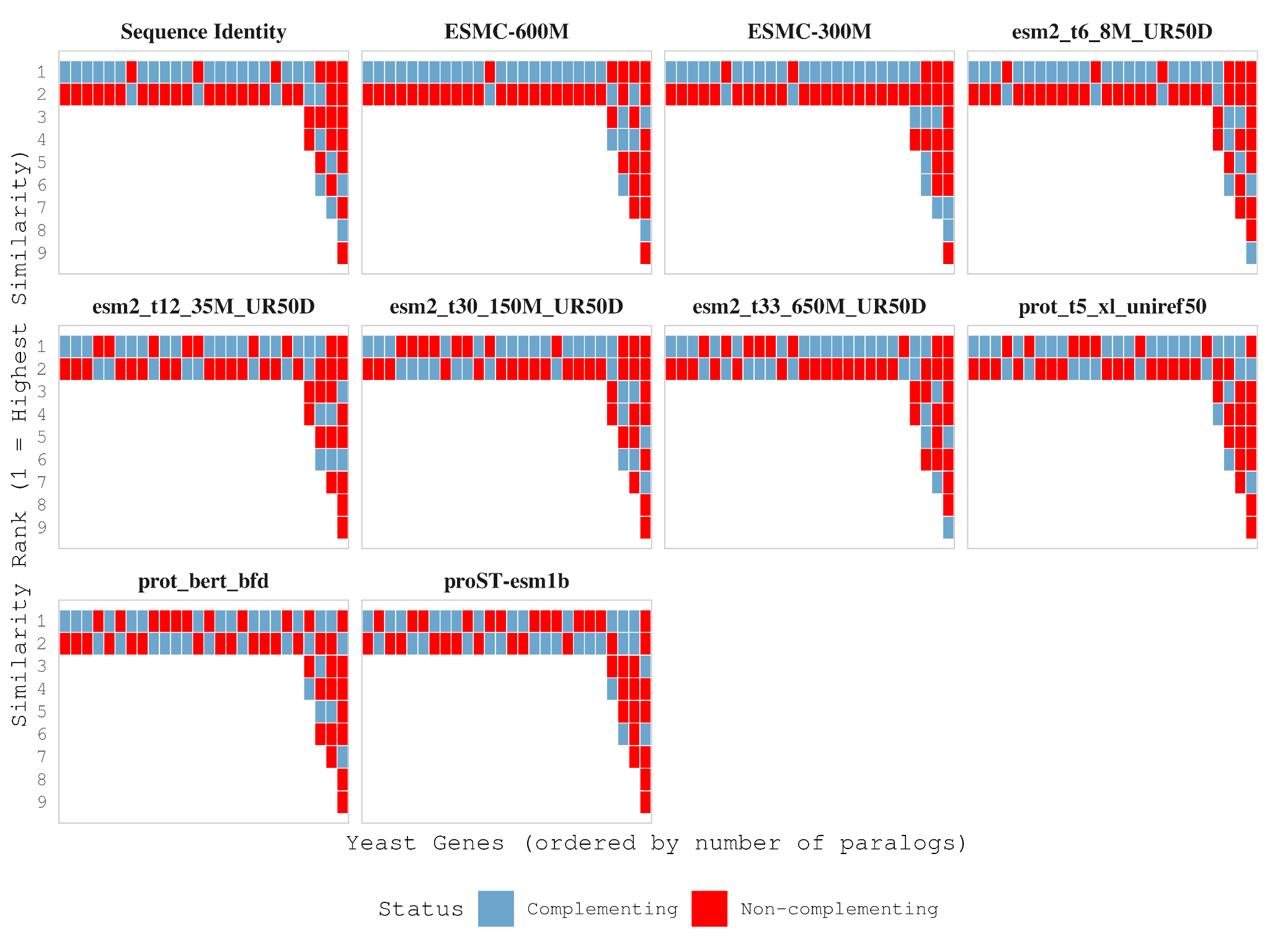
***Supplemental Figure 1. Poor performance prioritizing complementing proteins from one-to-many groups.*** *Both sequence identity and all models tested were unable to prioritize complementing genes from 1-to-many expanded families with a single yeast gene, but multiple human paralogs (In order:Uroporphyrinogen decarboxylase (4 human inparalogs), Myosin-1 (6 human inparalogs), Actin( 7 human inparalogs) and β-tubulin (9 human inparalogs)).*


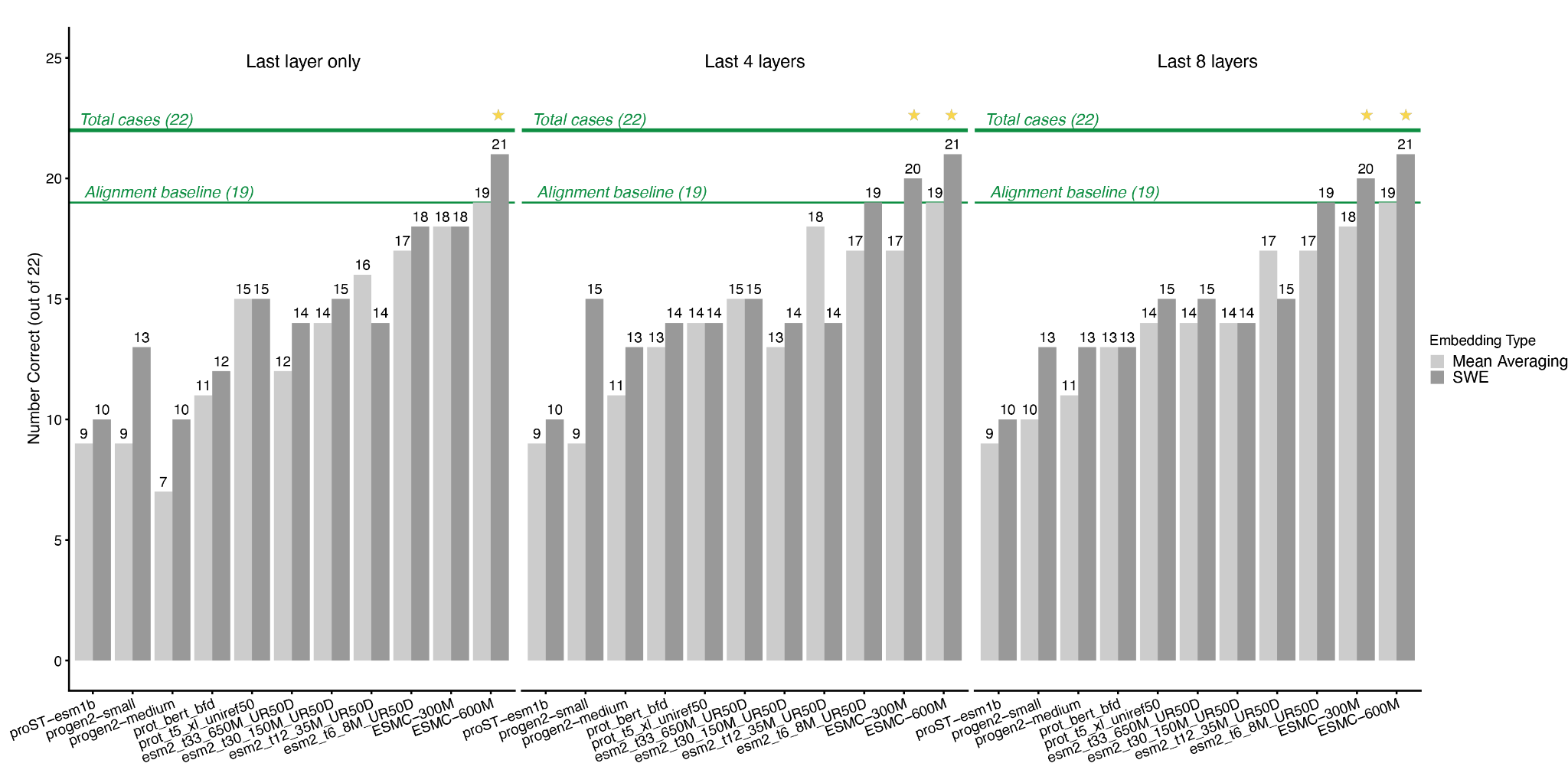


***Supplemental Figure 2. Comparison of different protein language models for predicting cross-species complementation between proteins with different layer choices.*** *Bar chart showing the predictive accuracy of various protein language models in identifying which human or Arabidopsis paralog can functionally complement a yeast ortholog. The horizontal line indicates baseline accuracy using sequence identity (19/22 correct). There is a general boost in accuracy from using concatenated embeddings from more than the last layer only. As esm2_t6_8M has only six layers, only six layers are used for the “Last 8 layers” panel.*
